# Effect of lupeol on testicular lesions induced by cadmium chloride in rats

**DOI:** 10.22038/AJP.2024.24716

**Published:** 2025

**Authors:** Parmida Arabkarami, Pejman Mortazavi, Saeed Hesaraki

**Affiliations:** 1 *Faculty of Specialized Veterinary Sciences, Science and Research Branch, Islamic Azad University, Tehran, Iran*; 2 *Department of Pathology, Faculty of Specialized Veterinary Sciences, Science and Research Branch, Islamic Azad University, Tehran, Iran*

**Keywords:** Infertility, Lupeol, Cadmium chloride, Malondialdehyde, Superoxide dismutase

## Abstract

**Objective:**

Heavy metals and environmental pollutants, such as cadmium chloride, congenital disorders, and certain diseases, can lead to infertility in men. In this study, the effects of lupeol (an active pentacyclic triterpenoid with antioxidant properties) on testicular injuries induced by cadmium chloride were investigated in male rats.

**Materials and Methods:**

Lupeol was obtained from Sigma-Aldrich, and the experiment included 40 male Wistar rats divided into 8 groups (healthy control, healthy rats treated with 100, 200, and 400 mg/kg of lupeol, cadmium chloride, and three groups that received cadmium chloride and were treated with 100, 200, and 400 mg/kg of lupeol). After oral treatment, rats were anesthetized, and blood and testicular tissue sampling was done. Subsequent analysis of oxidative stress enzymes, such as malondialdehyde (MDA) and superoxide dismutase (SOD), testosterone, sperm motility, and Aquaporin 9 (AQ9) levels was performed using ELISA, histopathology, and immunohistochemistry techniques, respectively. Immunohistochemistry staining was done for expression of Aquaporin 9 in seminiferous tubules.

**Results:**

The results showed that compared to the healthy control, cadmium chloride caused a significant decrease in SOD, testosterone, sperm motility, and sperm vitality, with severe destruction of spermatogenic tubes and a significant increase in MDA and AQ9 rate. Rats which were treated with cadmium chloride+400 mg/kg of lupeol showed a significant increase in SOD, testosterone, histopathology, sperm motility, and sperm vitality rate, and significant organization of spermatogenic tubes in testis tissue. There was also a decrease in MDA and AQ9 of rats that received high-dose lupeol. (p<0.001)

**Conclusion:**

This study suggests that lupeol has a high potential for improving male reproduction and antioxidants in rats exposed to oxidative stress.

## Introduction

Infertility is a condition of the reproductive system in which couples of reproductive age cannot conceive after trying for 12 months or more without using protection. (Deyhoul et al., 2017)

According to the World Health Organization, it is a significant health and social concern affecting men and women.(WHO, 2024) On the other hand, our environment is full of chemicals and pollutants that can harm human health (such as heavy metal salts, radioisotopes, and toxic substances released into the air by factories) (Alengebawy et al., 2021; Khatun et al., 2022)

Exposure to these pollutants can increase the number of oxidants, such as free radicals, in the body which can have harmful effects on sperm function in men (Nateghian and Aliabadi, 2020). Increases in free radicals and lipid peroxidation levels can lead to oxidative stress which is the main cause of functional damage and decreased sperm quality. This condition is closely associated with infertility in males (Bansal and Bilaspuri, 2011). 

Enzymatic changes and damage to sperm cells are important factors contributing to male infertility (El-Tohamy, 2012). During spermatogenesis, DNA damage caused by the destructive effects of free radicals, is the most significant factor that leads to male infertility. During spermatogenesis, sperm cells lose a considerable amount of their cytoplasm and antioxidant substances, which makes them vulnerable to oxidative stress (El-Tohamy, 2012). However, sperm fluid contains many antioxidants such as ascorbic acid, catalase, superoxide dismutase (SOD), and urates (Eid Hammadeh et al., 2009). Oxidative stress leads to fat oxidation and production of malondialdehyde (MDA), which is the main indicator of oxidative stress and defective sperm function. Recent studies have shown that infertile males have lower levels of antioxidants in their plasma than fertile males (Lykkesfeldt, 2007).

Despite the presence of antioxidant substances in semen, it seems that these antioxidants cannot protect spermatogenic tubes properly against harmful factors, and therefore, external sources of antioxidants are required to reduce the severity of these damages (Ribeiro et al., 2021).

Many studies are related to investigating plants' healing properties for various diseases (Agarwal et al., 2021; Jarema, 2008; Moghaddam et al., 2016). One of these plants is *Alhagi maurorum *(Ahmad et al., 2010). Lupeol, a triterpene found in various vegetables and plants, is the active ingredient in *Alhagi maurorum*. This compound has been shown to have many valuable diseases effects, including preventative, therapeutic, and improving factors for various disorders. Lupeol has a significant impact on molecular pathways that are connected with nuclear factor kappa B (NFκB), Fas, Kras, phosphatidylinositol-3-kinase (PI3 K)/Akt, and Wnt/β-catenin in a wide range of cells (Saleem, 2009). 

Lupeol is a triterpenoid that indicates different biological activities, such as anti-inflammatory, antioxidant, antidiabetic, anti-arthritic, anti-mutagenic and anti-malarial features (Chaturvedi et al., 2008). It has antibacterial, anti-inflammatory, antipyretic, analgesic, antioxidant, digestive, cardiovascular, diuretic, and repairing tissue effects (Al-Snai et al., 2019; Muhammad et al., 2015; Sheweita et al., 2016).

Recent research has shown that treatment with lupeol significantly increased the number of healthy sperm and GSH (Glutathione) levels in unfertilized rats induced by CdCl2. In addition, there was a significant decrease in the percentage of dead and abnormal sperm and MDA levels compared to the control group (Ahmed, 2019). Since the plant extract was investigated in Ahmed 2019 study, we decided to study the role of lupeol as the active substance of *Alhagi maurorum* and investigate the changes in aquaporin 9 which is a water-selective membrane channel in seminiferous tubules. Therefore, in the present study, the effect of lupeol on cadmium-related testicular damage in adult male Wistar rats was assessed.

## Materials and Methods

### Material purchase

Lupeol (product number: L5632) was bought from Sigma-Aldrich, Germany. This compound is hydrophobic and according to Sigma-Aldrich’s instructions it should be dissolved in ethanol. It was well reported that ethanol is an effective solvent for solving antioxidant phenolic compounds (Marashdah and Al-Hazimi, 2010). Then, 1000 g of lupeol was mixed with 4000 ml of 96% ethanol to prepare the solution and then kept in the shaker for 48 hr; then, the solution was filtered by Whatman filter paper number 1. After the solving process, the obtained solution was kept in a closed container at 4°C until the experiments (Pereira Beserra et al., 2020).

### Experimental animals

Forty Wistar adult male rats (180-200 g) were purchased from Tehran University (Tehran, Iran) and randomly divided into 8 groups (n=5 per group). Rats care and the experimental steps were performed in full accordance with the criteria of care and use of institutional animals Medical Sciences Ethics Committee of Islamic Azad University of Science and Research Branch, Tehran, Iran with IR.IAU.SRB.REC.1401.331 ethical code (light/dark cycles (12.12 hr); temperature (23°C); and humidity (55%)).

### Animal study design

Treatment groups received Lupeol daily through oral gavage for 30 days. This study investigated the effects of different doses (100, 200, 400 mg/kg) of lupeol solution based on previous research on its LD50 (Jega et al., 2020).

1. Healthy control group: Served as the healthy control, and received physiologic saline orally for 30 days. 

2. Healthy experimental group 100: Served as a 100, and received 100 mg/kg of lupeol. 

3. Healthy experimental group 200: Served as a 200, and received 200 mg/kg of lupeol. 

4. Healthy experimental group 400: Served as a 400, and received 400 mg/kg of lupeol. 

5. Patient control group: Served as cadmium chloride, and received 3 mg/kg of cadmium chloride. 

6. Patient experimental group 100: Served as cadmium chloride+100, and received 3 mg/kg of cadmium chloride+100 mg/kg of Lupeol. 

7. Patient experimental group 200: Served as cadmium chloride+200, and received 3 mg/kg of cadmium chloride+200 mg/kg of Lupeol. 

8. Patient experimental group 400: Served as cadmium chloride+400, and received 3 mg/kg of cadmium chloride+400 mg/kg of Lupeol.

### Induction of infertility through cadmium chloride

 For induction of infertility, the cadmium chloride, cadmium chloride+100, cadmium chloride+200, and cadmium chloride+400 groups received 1 ml of cadmium chloride (intraperitoneally for 30 days). 

### Surgical procedures

After the end of the treatment period with lupeol, all rats in the groups were euthanized using Pentobarbital (150 mg/kg IP, intraperitoneal). Then, the testicles of the rats were removed through dissection. After that, two longitudinal and transverse slits were created in the lower abdomen to remove the testis and examine the position of the testicles, and the slits were sutured. Finally, the testicles of adult rats were cautiously taken out of the body, and tissue preparation was carried out.

### Blood sampling

 The rats were first anesthetized to obtain blood serum, and blood was drawn from their hearts using a 3 ml syringe. The blood sample was then transferred into sterile microtubes and centrifuged at 2000 rpm for 5 min. The resulting blood serum was carefully separated into a new microtube and stored for further analysis.

### Tissue preparation

One testis was fixed in Bouin solution for 24 hr for histopathological analysis using H&E (Haematoxylin and Eosin) staining. Another testis was homogenized and used for MDA and SOD determination.

### Oxidative stress analysis

The tissue MDA level was determined by a method based on the reaction with thiobarbituric acid (TBA). In the TBA test reaction, MDA or MDA-like substances and TBA react. The reaction causes a pink pigment with maximum absorption at 532 nm. The SOD activity is expressed as nmol/g tissue. Tissue SOD activity was measured according to the method of Paoletti and Mocali (Paoletti et al., 1986). In brief, the superoxide anions were generated from manganese (II) chloride and mercaptoethanol in the presence of acidethylenediaminetetraacetic acid. The SOD level was determined based on its ability to inhibit nicotinamide adenine dinucleotide oxidation in the reaction mixture after the addition of tissue homogenate. Nicotinamide adenine dinucleotide oxidation was measured at 340 nm. The SOD activity is expressed as U/mg tissue. Assay kits for MDA and SOD were purchased from Randox (Randox Laboratories Ltd., Crumlin, Antrim, United Kingdom) and evaluated using an ELISA reader (Dana, Iran).

### Testosterone assay

For testosterone assay, the Rat Testosterone ELISA kit manufactured by CUSABIO was used. 96-well pellets coated with antibody against estradiol were prepared. Then, rats serum samples standard and control samples were added to the wells. In the next phase, HPR conjugate was added to all the wells. The plate was covered with a cover and incubated for 2 hr at 37^o^C. Then, the wells were washed three times using a plate washer. After the last washing and ensuring there was no washing solution in the wells, the TMB, tetramethylbenzidine substrate solution was added to all the wells, and the plate was incubated for 30 min at room temperature in the dark. Then, to stop the reaction, a stop solution was added, the color change from blue to yellow was observed, and the light absorbance and the concentration of estradiol in the sample were read (450±2 nm) with an ELISA reader.

### Sperm analysis

Computer-aided sperm analysis was used to measure sperm movements in different directions. Sperm samples were separated from other areas of the epididymis during surgery and placed in a culture medium containing serum. In the next step, they were checked with the sperms with the KASA machine. According to the protocol approved by the WHO, the sperms were classified into four groups: A, B, C, and D (Progressive, Non-Progressive, and Immotile) based on the calculated velocities (WHO, 2021). Group A are sperms that are fast and progressive. Group B sperms are also progressive sperms but at a lower speed than group A. Group C are motile sperm whose movement is not developed and moves in situ or has spiral and rotary motion. Group D are non-motile sperm.

### Histopathological examination

The tissue was fixed in Bouin solution and embedded in paraffin blocks. A tissue section (6 μm) was obtained, deparaffinized, and stained with Hematoxyline and Eosin. The testicular tissue was evaluated in random order with standard light microscopy by an observer who was unaware as to which group the rat belonged. The testis sections were graded numerically to assess the degree of histological changes associated with seminiferous tubule injury as previously described by Johnsen scoring as below (Johnsen, 1970): 

Score–10: Complete spermatogenesis with many spermatozoa present. Score–9: Slightly impaired spermatogenesis with many late spermatids, disorganized epithelium. Score–8: Less than five spermatozoa per tubule, few late spermatids. Score–7: No spermatozoa, no late spermatids, many early spermatids. Score–6: No spermatozoa, no late spermatids, few early spermatids. Score–5: No spermatozoa or spermatids, many spermatocytes. Score–4: No spermatozoa or spermatids, few spermatocytes. Score–3: Spermatogonia only. Score–2: No germinal cells, Sertoli cells only. Score–1: No seminiferous epithelium

### Immunohistochemistry

The immunohistochemical technique was used to evaluate the expression of the aquaporin 9 protein, and this technique was performed in 6 steps (tissue preparation, antigen retrieval, blocking, immune-staining, counter-staining, and mounting). To check the expression of Aquaporin in the samples, the stained slides were studied by light microscope; ten fields were randomly selected under the microscope; in each slide, 100 spermatocyte cells were counted, and the immunoreactive nuclear ratio was obtained. The percentage of stained nuclei among 1000 cells was determined at the end. Immunoreactivity below 5% was considered negative, and above 5% was considered positive. The negative control was the staining of slides without secondary antibodies (<5%=0; 5%-30%=1; 30%-60%=2; >60%=3).

### Statistical analysis

Data is reported as mean±SD and the graphs were plotted using the GraphPad Prism version8. Data were analyzed using analysis of statistical variances (ANOVA) followed by Tukey *post hoc* test and a p-value less than 0.05 was considered a significant difference.

## Results

**Figure 1:**
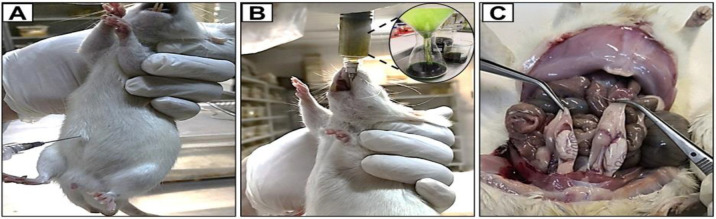
Infertility was induced in rats through cadmium chloride injection (A). Rats were then treated with varying doses of lupeol via gavage for 30 days (B). At the end of the treatment period, testis tissue samples were collected (C).

Male rats rendered infertile by cadmium chloride (Figure 1A) were treated with different doses of lupeol for 30 days (Figure 1B); the animals were sampled to assess the effect of the lupeol on testicular injury induced by cadmium chloride (Figure 1C).

### Oxidative stress

At the end of the study period, the group treated with cadmium chloride showed a significant decrease in SOD (p<0.0001) and an increase in MDA level (p<0.0001) as compared to the healthy control group. Additionally, the groups treated with cadmium chloride+100, cadmium chloride+200 mg/kg, and cadmium chloride+400 mg/kg showed a high level of 

**Figure 2:**
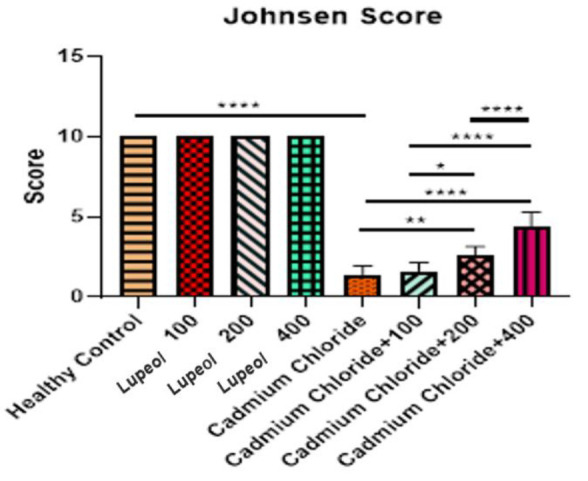
Effect of 100, 200, and 400 mg/kg of lupeol, cadmium chloride, cadmium chloride+100, cadmium chloride+200 mg/kg, and cadmium chloride+400 mg/kg of lupeol after 30 days on tissue values of superoxide dismutase (SOD) (A) and malondialdehyde (MDA) (B); values are expressed as means±SE from 5 rats per group; (*p<0.05, ***p<0.001, and ****p<0.0001)

SOD and low level of MDA (p<0.0001) as compared to the group treated with cadmium chloride alone. The results obtained from comparing cadmium chloride+400 with cadmium chloride+100 and cadmium chloride+200 showed that the amount of SOD increased significantly (p<0.0001), while the amount of MDA decreased significantly (p<0.001 and p<0.0001 for X and Y, respectively.) (Figure 2). 

### Testis histopathological examination

**Figure 3:**
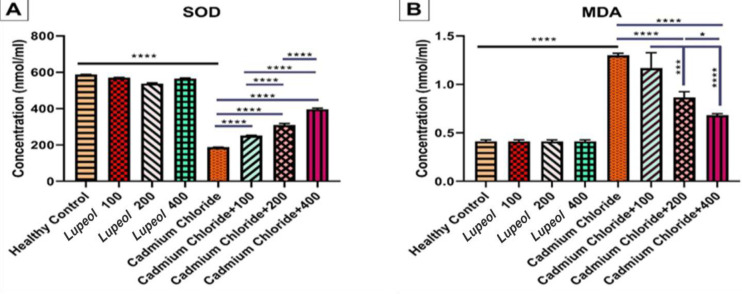
Effect of 100, 200, and 400 mg/kg of Lupeol, cadmium chloride, cadmium chloride+100, cadmium chloride+200 mg/kg, and cadmium chloride+400 mg/kg of lupeol after 30 days on testis tissue histopathology Johnsen score; values are expressed as means±SE from 5 rats per group; (*p<0.05, **p<0.01, and ****p<0.0001).

Histopathology evaluation (based on Johnsen score**(** as shown in Figure 3, the group treated with cadmium chloride exhibited a significant decrease in spermatogenesis compared to the healthy control (p<0.0001). However, the groups cadmium chloride+200 mg/kg of Lupeol (p<0.01) and cadmium chloride+400 mg/kg of Lupeol (p<0.0001) showed increases in sperm production compared to cadmium chloride. On the other hand, a significant increase in producing sperms was observed in cadmium chloride+400 compared to cadmium chloride+100 and cadmium chloride+200 (p<0.0001).

**Figure 4:**
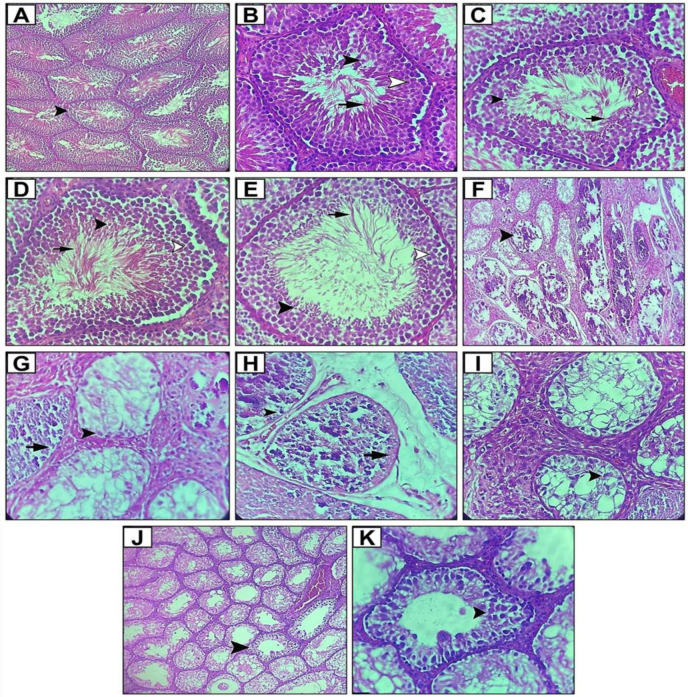
Light microscopy images of testis sections, hematoxylin and eosin (H&E) staining of: cross-section of the testis in the healthy control group (the arrowhead shows the healthy sperm tubes) (magnification x100) (A), cross-section of the testicular spermatogenic tube in a healthy control group with spermatocyte cells (white arrowhead), a large number of spermatids (black arrowhead) and spermatozoa (arrow) (magnification x400) (B), cross-section of the testicular spermatogenic tube in the group treated with 100 mg/kg Lupeol* um *with spermatocyte cells (white arrowhead) and a large number of spermatids (black arrowhead) and spermatozoa (arrow) (magnification x400) (C), cross-section of testicular spermatogenic tube in the group treated with 200 mg/ lupeol with spermatocyte cells (white arrowhead) and a large number of spermatids (black arrowhead) and spermatozoa (arrow) (magnification x400) (D), cross-section of testicular spermatogenic tube in the group treated with 400 mg/kg Lupeol with spermatocyte cells (white arrowhead) and a large number of spermatids (black arrowhead) and spermatozoa (arrow) (magnification x400) (E), cross-section of the testis in the group treated with cadmium chloride with severe destruction of spermatogenic tubes (arrow) (magnification x100) (F), cross-section of the testicular spermatogenic tube of the cadmium chloride-treated group with few tuberculous masses (arrowheads) in one tube and no tuberculous masses in the other tube (arrow) (magnification x400) (G), the cross-section of a testicular spermatogenic tube in the cadmium chloride+100 mg/kg shows a tuberculous mass (arrowhead) in one tube, and no cells can be seen in the other tube (arrow) (magnification x400) (H), a cross-section of a testicular spermatogenic tube in the cadmium chloride+200 mg/kg with few spermatocytes (arrowhead) (magnification x400) (I), cross-section of the testis in the in the cadmium chloride+400 mg/kg with significant organization of spermatogenic tubes (arrowhead) (magnification x100) (J), cross-section of a testicular spermatogenic tube in the cadmium chloride+400 mg/kg with many spermatocytes (arrowheads) (magnification x100) (K).

Interaction between lupeol and sex hormones may significantly impact spermatogenesis, as shown in Figure 4. As reflected by histopothology which is defined as Johnsen score, increasing the solution dosage enhances spermatogenesis.

### Evaluation of AQ9 score in rat

**Figure 5:**
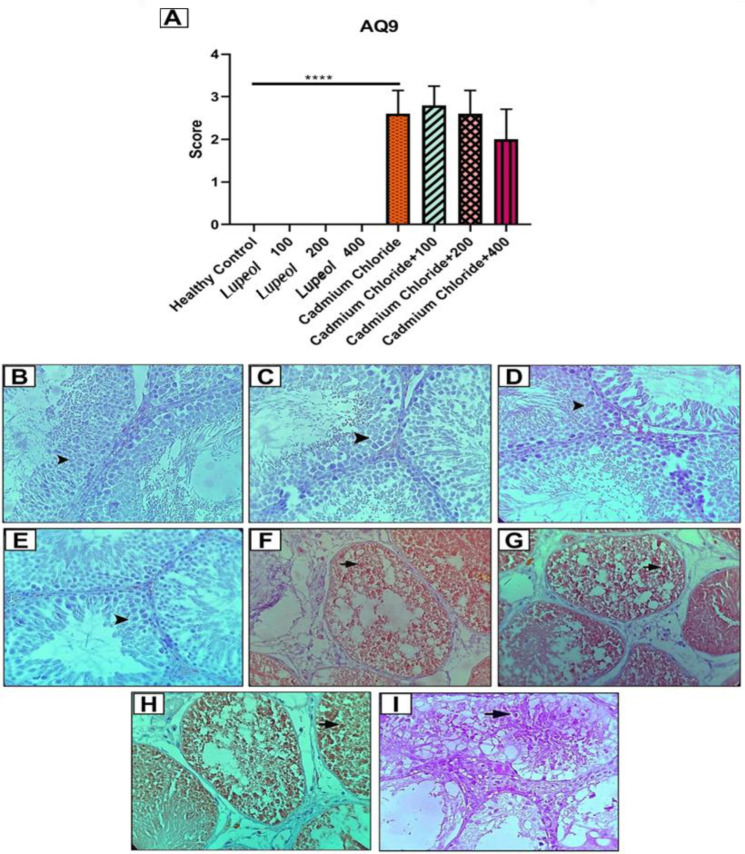
Effect of 100, 200, and 400 mg/kg of lupeol, cadmium chloride, cadmium chloride+100, cadmium chloride+200 mg/kg, and cadmium chloride+400 mg/kg lupeol after 30 days on rat's testis AQ9 expression score; value were expressed as means ± SE from 5 rats per group; (****p<0.0001). Light microscopy images of AQ9 score on testis sections, hematoxylin and eosin (H&E) staining of: cross-section of the testis in the healthy control (B), 100 (C), 200 (D), and 400 (E) mg/kg of Lupeol, cadmium chloride (F), cadmium chloride+100 (G), cadmium chloride+200 mg/kg (H), and cadmium chloride+400 mg/kg (I) of Lupeol after 30 days. (Magnification x400)

In analyzing the AQ9 score at the end of the treatment period, it was observed that the rate of AQ9 in the group treated with cadmium chloride decreased significantly compared to the healthy control group (p<0.0001), as shown in Figure 5A. In a cross-section of a testicular spermatogenic tube in the healthy control group, the absence of AQ9 expression was seen in spermatocytes (arrowhead), as shown in Figure 5B. In the groups treated with 100 (Figure 5C), 200 (Figure 5D), and 400 mg/kg (Figure 5E) of lupeol, the absence of AQ9 expression was also observed in spermatocytes (arrowhead). However, in the cross-section of the spermatogenic tube of the testis under treatment with cadmium chloride, there was a strong expression of AQ9 in spermatocytes (arrow), as shown in Figure 5F. In the cross-section of a testicular spermatogenic tube in cadmium chloride+100 (Figure 5G), cadmium chloride+200 (Figure 5H), and cadmium chloride+400 (Figure 5I), there was a high rate of AQ9 expression observed in spermatocytes (arrowhead).

### Evaluation of testosterone levels in rats

**Figure 6:**
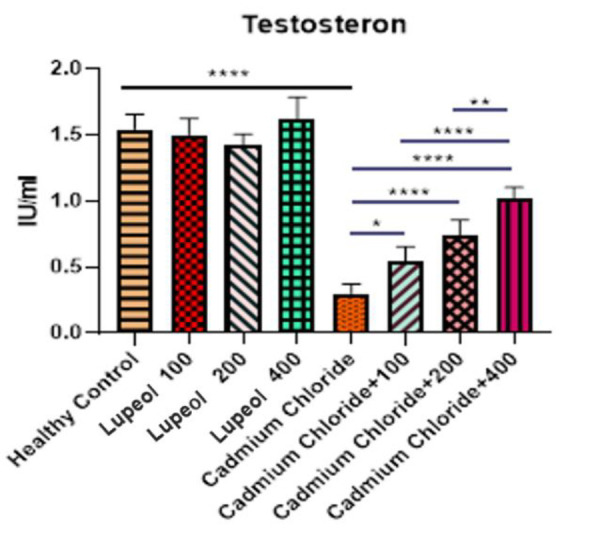
Effect of 100, 200, and 400 mg/kg of lupeol, cadmium chloride, cadmium chloride+100, cadmium chloride+200 mg/kg, and cadmium chloride+400 mg/kg of lupeol after 30 days on testosterone rate (IU/ml) of rats; values are expressed as means±SE from 5 rats per group; (*p<0.05, **p<0.01, and ****p<0.0001).

The testosterone levels were analyzed in the different groups, and the findings are presented in Figure 6. As per the results, the group treated with cadmium chloride had a lower level of testosterone than the healthy controls (p<0.001). However, the groups that were treated with cadmium chloride+100, cadmium chloride+200, and cadmium chloride+400 demonstrated a significant increase compared to the group treated with cadmium chloride alone (p<0.05, p<0.001, and p<0.0001, respectively). 

### Evaluation of sperm motility percentage in rats

**Figure 7:**
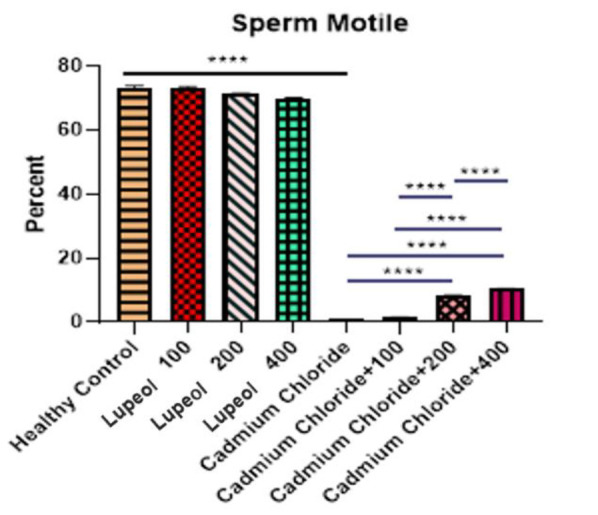
Effect of 100, 200, and 400 mg/kg of lupeol, cadmium chloride, cadmium chloride+100, cadmium chloride+200 mg/kg, and cadmium chloride+400 mg/kg of lupeol after 30 days on sperm motility percentage of rats; values are expressed as means±SE from 5 rats per group. (****p<0.0001).

The motility of the sperm was analyzed in the groups under study, and the results are depicted in Figure 7. The findings revealed that the group treated with cadmium chloride had a substantially reduced percentage of motile sperm compared to the healthy control group (p<0.0001). Conversely, the groups treated with cadmium chloride+200 and cadmium chloride+400 exhibited an increase in significance compared to group 5. (patient control group) p<0.0001). Moreover, the group treated with cadmium chloride and 200 mg/kg of lupeol showed a significant increase in sperm motility compared to the group treated with cadmium chloride+100. The patient experimental group 400 that was served cadmium chloride and 400 mg/kg of lupeol showed the highest improvement in sperm motility.

## Discussion

Male infertility is often caused by insufficient production of healthy and active sperm. Investigating the factors and causes of male infertility and ways to prevent it is essential. Oxidative stress can negatively impact the production and count of sperm in men, leading to infertility. This occurs due to the production of free radicals in the testicular tissue. However, the use of antioxidants has been proven effective in controlling and treating this problem by reducing the damage caused by free radicals, strengthening the testicular blood barrier, and protecting and repairing sperm DNA (Hamid et al., 2018). In a recent study, lupeol was used in an experimental testicular torsion model, and it was a preventing agent for prevention of testicular ischemia. In addition to the damage-reducing and protective effects of lupeol, its anti-inflammatory, antioxidant, and anti-apoptotic effects were demonstrated (Azzam et al., 2024).

Therefore, the current study examined how treating the testis with lupeol affects the testis structure, spermatogenesis, and oxidative stress parameters.

According to a study, lupeol is one of the main ingredients that can be found in triterpenoids in *Salvia* species, and due to its antioxidant features, it can be used as an anti-inflammatory factor for damaged tissue (Topçu, 2006).

A recent study has shown that lupeol can inhibit the growth of A427 lung cancer in humans and has antiproliferative effects due to apoptosis induction. Lung cancer is one of the most malignant cancers diagnosed in developed countries with high rates of morbidity and mortality. However, lupeol, being an antioxidant, can reduce the harmful effects of cancer cells and induce potent and selective cytotoxic effects and these effects were mediated via apoptosis, ROS generation, loss of MMP and inhibition of mTOR/PI3K/AKT signalling pathway (He et al., 2018). 

In a study conducted on Wistar rats, researchers discovered in the cadmium-intoxicated rats, there was a significant increase of MDL release, both under basal and induced conditions. Since lupeol has been shown to exhibit anti-inflammatory and anti-urolithiasis activities in rats, it is used for hepatoprotective effects in the liver. However, the adverse effects were reduced by lupeol, a potent flavonoid with antioxidant properties. The oral administration of lupeol scavenges the free radicals and adverse effects of CdCl2 by improving the antioxidant status of the liver (Nna et al., 2017).

Another recent study has illustrated the significant role of *Cichorium intybus* extract, which can lead to a reduction in free radicals, apoptosis, and a good improvement in antioxidant status (Hashemi et al., 2023). 

Lupeol has a strong effect on cancer, as well as a great impact on SOD and MDA. It can be recognized as an anti-cancer factor by inducing oxidative stress, apoptosis and anti-inflammatory agent via the AMPK/NF-κB pathway in nasopharyngeal carcinoma (Zhou et al., 2022).

It has been found that Lupeol has a high potential in hepato-neurotoxicity and neurobehavioral changes caused by leads in rats. It removes oxidative stress and the caspase-3-dependent apoptosis pathway (Babu et al., 2019).

Researchers found that lead increases motor activities and sensorimotor deficits while decreasing dopamine levels, which can harm the liver by disturbing its function. It also causes difficulty in the secretion of essential enzymes, which leads to severe tissue changes. Other effects of lead exposure are cell apoptosis, an increase in oxidative stress, and a decrease in MDA and GSH levels. However, after treating the animals with Lupeol, the toxic effects of lead were significantly diminished. Lupeol can also protect liver and brain tissues against lead by reducing apoptosis and oxidative stress in mice (Saber et al., 2022).

After conducting experiments on mice, it has been found that Lupeol can protect their livers from damage caused by carbon tetrachloride. The group of mice that were given the extract showed significant changes in various indicators such as total cholesterol, triglycerides, lipoprotein fraction, alanine aminotransferase, aspartate aminotransferase, alkaline phosphatase, total protein, albumin, total bilirubin and oxidative stress markers like glutathione, MDA, glutathione peroxidase, SOD, catalase. The results indicated that the extract significantly affects hypolipidemia, liver health, and antioxidant effects in mice exposed to carbon tetrachloride (M El Mallah, 2016).

An increase in MDA level and a decrease in SOD can cause lipid peroxidation, which in turn can damage tissues and compromise the body's natural antioxidant defense mechanism, leading to the formation of excessive free radicals. Our findings, like previous studies, indicate that Lupeol contains numerous flavonoids, such as tamarixetin 3-O-dirhamnoside, quercetin 3-O-rhamnoside, and isorhamnetin. As a result, this plant plays a significant role in traditional medicine. It treats rheumatic pains, bilharzia, liver disorders, urinary tract infections, and digestive disorders. Additionally, at a 400 mg/kg dosage, Lupeol exhibits peripheral and central analgesic and antioxidant activity (Atawodi, 2005). Our results showed that compared to the healthy control, cadmium chloride caused a significant decrease in SOD, testosterone, sperm motility, and sperm vitality, with severe destruction of spermatogenic tubes and a significant increase in MDA and AQ9 rate (p*<*0.0001). After treating the rats with cadmium chloride+400 mg/kg of Lupeol, the results showed a significant increase in SOD, testosterone, Johnsen score, sperm motility, sperm vitality rate, and significant organization of spermatogenic tubes in testis tissue. There was also a decrease in MDA and AQ9 rates compared to the healthy control, healthy rats treated with 100, 200, and 400 mg/kg of Lupeol, cadmium chloride, and the groups that received cadmium chloride and treated with 100 and 200 mg/kg of Lupeol (p*<*0.001). This study suggests that Lupeol extraction has a high potential for controlling male reproduction and antioxidants in rats exposed to oxidative stress.

Among males who suffer from infertility, poisonous substances might be a strong agent inducing spermatogenesis problems. These dangerous chemicals including cadmium may lead to testicular malfunction and disruption in healthy production of sperm. In addition to that, toxic chemicals’ exposure to nature causes an excessive increase of free radicals solutions and oxidant compounds. Therefore, using active ingredients from medicinal plants, vegetables, and fruits might be a perfect solution as a barrier for oxidant compounds. The results indicate that Lupeol reduces the toxic effects of cadmium chloride effectively and can be used as a suitable medicine for fertility with fewer side effects rather than other chemical medicines.
